# Synergy of TLR3 and 7 ligands significantly enhances function of DCs to present inactivated PRRSV antigen through TRIF/MyD88-NF-κB signaling pathway

**DOI:** 10.1038/srep23977

**Published:** 2016-04-05

**Authors:** Yue Hu, Xiaoyan Cong, Lei Chen, Jing Qi, Xiangju Wu, Mingming Zhou, Dongwan Yoo, Feng Li, Wenbo Sun, Jiaqiang Wu, Xiaomin Zhao, Zhi Chen, Jiang Yu, Yijun Du, Jinbao Wang

**Affiliations:** 1 Key Laboratory of animal biotechnology and disease control and prevention of Shandong Province, College of Animal Science and Veterinary Medicine, Shandong Agricultural University, Tai’an 271018, China; 2 Shandong Key Laboratory of Animal Disease Control and Breeding, Institute of Animal Science and Veterinary Medicine, Shandong Academy of Agricultural Sciences, Sangyuan Road No. 8, Jinan 250100, China; 3 Department of Pathobiology, University of Illinois at Urbana-Champaign, 2001 South Lincoln Ave, Urbana, IL 61802, USA; 4 Department of Biology and Microbiology, Department of Veterinary and Biomedical Sciences, South Dakota State University, Brookings, SD, 57007, USA

## Abstract

PRRS is one of the most important diseases in swine industry. Current PRRS inactivated vaccine provides only a limited protection and cannot induce sufficient cell-mediated immune responses. In this study, we first found that the mRNA and protein levels of Th1-type cytokines (IFN-γ, IL-12) and Th2-type cytokines (IL-6, IL-10) were significantly increased through TRIF/MyD88-NF-κB signaling pathway when porcine peripheral blood monocyte-derived dendritic cells (MoDCs) were treated with poly (I: C) of TLR3 ligand and imiquimod of TLR7 ligand, along with inactivated PRRSV antigen. Meanwhile, the ability of catching PRRSV antigen was also significantly enhanced. In mice experiment, it was found that the PRRSV-specific T lymphocyte proliferation, the percentages of CD4^+^, CD8^+^ T lymphocytes and PRRSV-specific CD3^+^ T cells producing IFN-γ and IL-4, the levels of Th1- and Th2-type cytokines and the titers of neutralization antibody were significantly enhanced in poly (I: C), imiquimod along with inactivated PRRSV group. Taken together, results of our experiments described for the first time that synergy of TLR3 and 7 ligands could significantly enhance the function of DCs to present inactivated PRRSV antigen through TRIF/MyD88-NF-κB signaling pathway and be used as adjuvant candidate for the development of novel PRRS inactivated vaccine.

Porcine reproductive and respiratory syndrome (PRRS), characterized by reproductive failure in pregnant sows and gilts along with severe respiratory distress in piglets and growing pigs, is one of the most economically impacting diseases affecting the swine industry^1^,^2^. The causative agent is PRRS virus (PRRSV) in the family of *Arteriviridae*. PRRSV infects porcine alveolar macrophages (PAMs) and also replicates in MoDCs. The hallmarks of PRRS include weak immune response and persistent infection^3^,^4^,^5^,^6^. Current vaccines available for PRRS include live attenuated vaccine and inactivated vaccine. PRRS live attenuated vaccine is well recognized for its protective efficacy against PRRSV that is genetically homologous to the vaccine virus, but has a potential of reversion to virulence. PRRS inactivated vaccine, on the other hand, is well known for its safety, but cannot induce sufficient cellular immunity and only confers a limited protection. The use of appropriate immune adjuvant may help to enhance the efficacy of current PRRS inactivated vaccine^7^.

The mammalian immune system is comprised of two functional branches: innate immunity and adaptive immunity. The innate immune system recognizes microorganisms via a limited number of pattern recognition receptors (PRRs)^8^. As host PRRs, Toll-like receptors (TLRs), play a key role in the recognition of microbial pathogen associated molecular patterns (PAMPs) and trigger the activation of specific signaling pathways, thereby inducing the transcription of inflammatory and/or anti-inflammatory cytokines^9^,^10^,^11^. TLRs are differentially expressed in immune cells, and preferentially expressed in antigen-presenting cells (APCs) such as monocyte/macrophages and dendritic cells (DCs). DCs play a critical defense role through integrating and coordinating the innate and adaptive components of the immune system, thereby directing the appropriate immune responses against infectious agents^10^,^12^,^13^,^14^.

When a TLR ligand binds to the receptor, an intracellular signal transduction cascade is triggered, altering the pattern of gene expression in the cells. Many adjuvants are believed to be mimics of TLR ligands and TLRs turn out to be important for immune responses to vaccines as well as natural infection^3^. TLR3 is triggered by double-stranded RNA (dsRNA) that is produced during the replication of most viruses. Poly (I: C), a synthetic analog of dsRNA, is the ligand of choice for TLR3. The communication of TLR3 with dsRNA triggers a TRIF-dependent signaling cascade through the activation of NF-κB, MAP kinases and IRF3, and culminates in the production of inflammatory cytokines and type I IFNs^15^,^16^. TLR7 and TLR8 are also involved in responses to viral infections and recognize GU-rich, short single-stranded RNA as well as small synthetic molecules such as resiquimod, imiquimod and nucleoside analogues. Such recognitions lead to NF-κB activation through a MyD88-dependent pathway^17^,^18^,^19^. TLR3 and TLR7 signaling pathways involve different players but share some common regulators such as NF-κB between them^10^. NF-κB is a major transcription factor which functions on TLR signaling to control and elicit inflammation. NF-κB activity is found to be inducible in all cell types and it is now known that members of the NF-κB/Rel family regulate many genes involved in immune and inflammatory responses^20^,^21^,^22^,^23^.

Previous studies have shown that PRRSV infection increases the proportion of cells expressing TLR3 and TLR7^24^,^25^. In this study, we provide evidence that the synergistic combination of TLR3 and 7 ligands significantly enhances the function of MoDCs to present inactivated PRRSV antigen through TRIF/MyD88-NF-κB signaling pathway. The observed *in vitro* immune enhancing effect of the combination of TLR3 and 7 ligands is further confirmed in mice. These data offer insights to the mechanism evolved by the combination of TLR3 and 7 ligands to enhance the immune effects of inactivated PRRSV antigen.

## Results

### The mRNA and protein levels of cytokines in MoDCs stimulated with TLR ligands and inactivated PRRSV antigen

**Figure 1:**
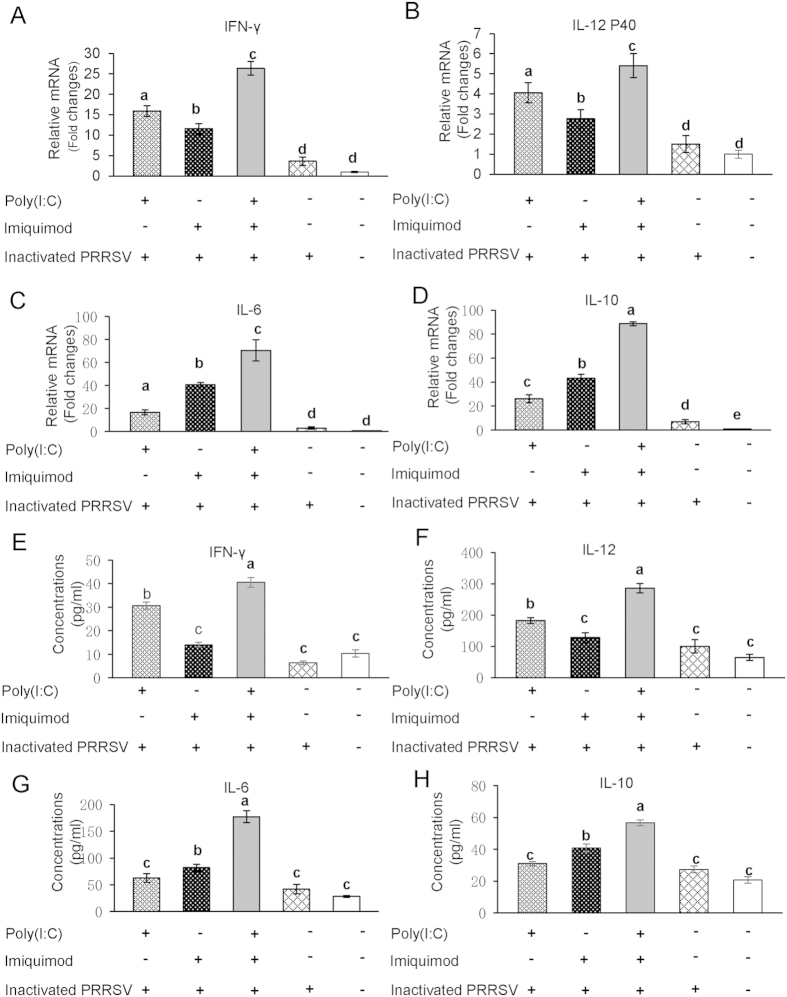
The mRNA and protein levels of cytokines in MoDCs stimulated with TLR ligands and inactivated PRRSV antigen. MoDCs were incubated with poly (I: C) and/or imiquimod along with inactivated PRRSV antigen for 12 h, the inactivated PRRSV antigen and RPMI-1640 were used as control. The transcript levels of Th1-type cytokines IFN-γ (**A**) and IL-12 P40 (**B**), Th2-type cytokines IL-6 (**C**) and IL-10 (**D**) were analyzed by real-time RT-PCR. Relative transcript levels are shown as fold changes relative to the level for the control cells of the RPMI-1640 group. Concentrations of Th1-type cytokines IFN-γ (**E**) and IL-12 (**F**), Th2-type cytokines IL-6 (**G**) and IL-10 (**H**) in the supernatants of MoDCs were detected by ELISA. The data represent the means of three independent experiments, with each experiment performed in triplicate. Data represent the means ± standard deviations (error bars) of three independent experiments. Different letters (a–e) mean significant difference (*P* < 0.05).

MoDCs were stimulated with poly (I: C) and/or imiquimod along with inactivated PRRSV antigen for 12 h, the mRNA levels of Th1-type cytokines IFN-γ and IL-12 P40, Th2-type cytokines IL-6 and IL-10 were examined by real-time RT-PCR. As shown in Fig. 1A–D, MoDCs incubated with inactivated PRRSV antigen and RPMI-1640 control group showed a basal expression level of cytokines. The mRNA levels of Th1-type cytokines IFN-γ and IL-12 P40 were increased significantly in poly (I: C)-inactivated PRRSV antigen group than imiquimod-inactivated PRRSV antigen group (*P* < 0.05). In contrast, the mRNA levels of Th2-type cytokines IL-6 and IL-10 in imiquimod-inactivated PRRSV antigen group were higher than that observed in poly (I: C)-inactivated PRRSV antigen group (*P* < 0.05). Interestingly, we found that poly (I: C)-imiquimod-inactivated PRRSV antigen group showed the highest levels of both the Th1- and Th2-type cytokines (*P* < 0.05).

As shown in Fig. 1E–H, the concentrations of both Th1- and Th2-type cytokines were the highest in poly (I: C)-imiquimod-inactivated PRRSV antigen group (*P* < 0.05). Compared to inactivated PRRSV antigen and RPMI-1640 control group, the protein levels of Th1-type cytokines IFN-γ and IL-12 were higher in poly (I: C)-inactivated PRRSV antigen group, and Th2-type cytokines IL-6 and IL-10 were higher in imiquimod-inactivated PRRSV antigen group (*P* < 0.05). This tendency was consistent with the mRNA levels of cytokines in MoDCs.

### Activation of TRIF/MyD88-NF-κB signaling pathway in MoDCs treated with TLR3 and 7 ligands along with inactivated PRRSV antigen

**Figure 2:**
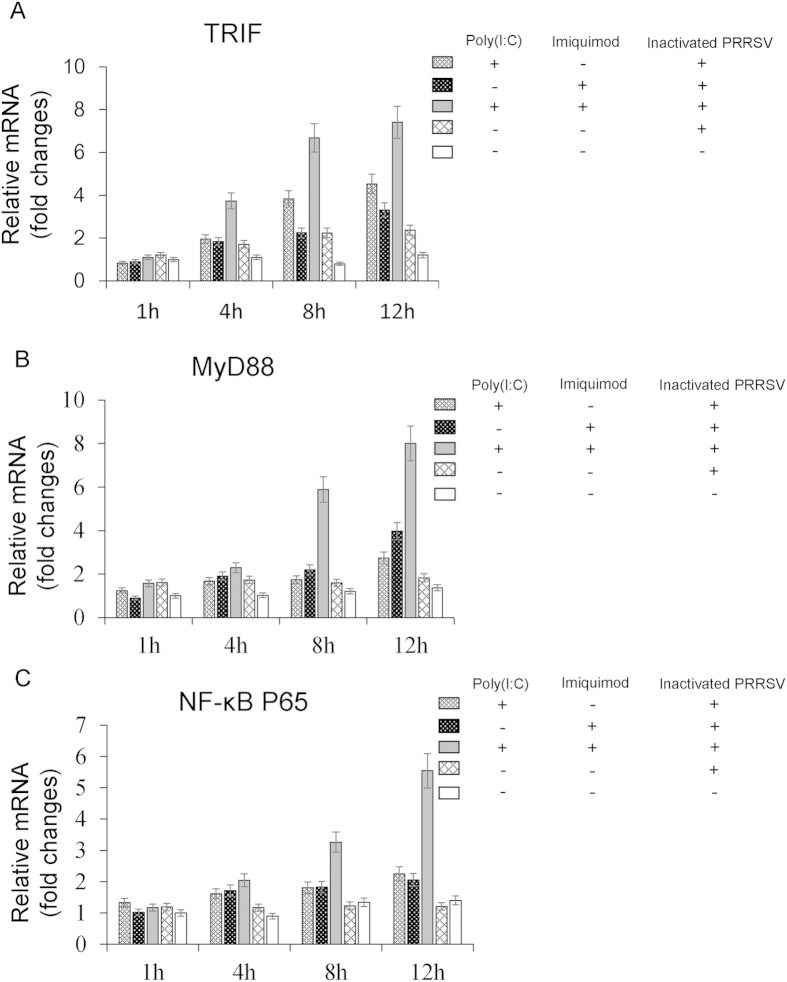
The mRNA levels of TRIF, MyD88 and NF-κB P65 in MoDCs stimulated with TLR ligands and inactivated PRRSV antigen for different times. MoDCs were incubated with poly (I: C) and/or imiquimod along with inactivated PRRSV antigen for 1 h, 4 h, 8 h and 12 h, the inactivated PRRSV antigen and RPMI-1640 were used as control. The mRNA levels of TRIF, MyD88 and NF-κB P65 were analyzed by real-time RT-PCR. Relative transcript levels are shown as fold changes relative to the level for the control cells of the RPMI-1640 group. The data represent the means of three independent experiments, with each experiment performed in triplicate. Data represent the means ± standard deviations (error bars) of three experiments.

TLRs and MyD88 are upstream regulatory factors of NF-κB^26^,^27^, and after stimulation with an appropriate ligand, TLRs relay the signal via MyD88. MyD88 is a common signal adaptor molecule shared by members of the TLR family except TLR3 which relays signals via TRIF to activate NF-κB^8^,^28^,^29^,^30^. When NF-κB is activated, inflammatory cytokines are produced^8^. To further investigate the pathways participating in the cytokines secretion, the mRNA levels of TRIF, MyD88, NF-κB P65 were measured in MoDCs stimulated with poly (I: C) and/or imiquimod along with inactivated PRRSV antigen for 1 h, 4 h, 8 h and 12 h by real-time RT-PCR. In inactivated PRRSV antigen and RPMI-1640 control groups, the mRNA levels of TRIF, MyD88, and NF-κB P65 remained at the basal level. In contrast, the mRNA level of TRIF was higher in poly (I: C)-inactivated PRRSV antigen group at 8 h and 12 h, and MyD88 was also higher in imiquimod-inactivated PRRSV antigen group at 12 h (Fig. 2A,B). However, the mRNA level of NF-κB P65 was not elevated in these two groups (Fig. 2C). Notably, the mRNA levels of TRIF and MyD88 were stimulated highest as early as 8 h, and the stimulation of NF-κB P65 was also the highest from 8 h in poly (I: C)-imiquimod-inactivated PRRSV antigen group (*P* < 0.05, Fig. 2A–C).

**Figure 3:**
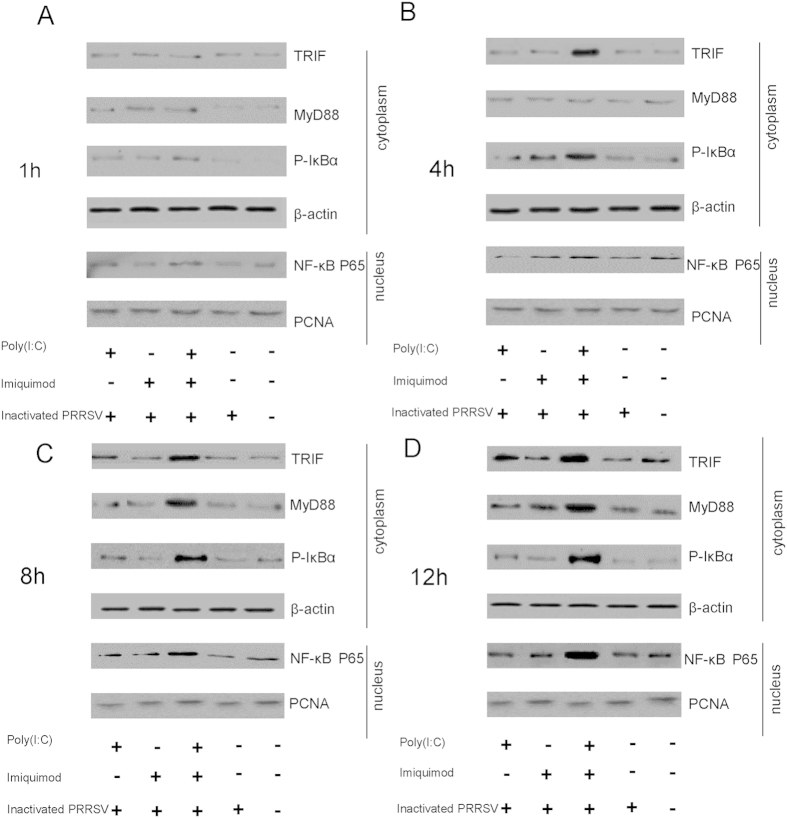
The protein levels of TRIF, MyD88, phospho-IκBα and NF-κB P65 in MoDCs stimulated with TLR ligands and inactivated PRRSV antigen for different times. MoDCs were incubated with poly (I: C) and/or imiquimod along with inactivated PRRSV antigen for 1 h, 4 h, 8 h and 12 h, the inactivated PRRSV antigen and RPMI-1640 were used as control. The protein levels of TRIF, MyD88, phospho-IκBα in cytoplasm and the protein levels of NF-κB P65 in nucleus were detected by western blotting. β-actin and PCNA were used as loading control in cytoplasm and nucleus, respectively. The data presented here are results from one experiment of three western blotting experiments.

The cytoplasmic protein levels of TRIF, MyD88 and phospho-IκBα and the nuclear protein levels of NF-κB P65 in MoDCs stimulated with poly (I: C) and/or imiquimod along with inactivated PRRSV antigen were measured for 1 h, 4 h, 8 h and 12 h by western blotting. In the cytoplasm, the protein levels of TRIF were increased in poly (I: C)-inactivated PRRSV antigen group at 8 h and 12 h, and MyD88 was stimulated higher in imiquimod-inactivated PRRSV antigen group at 12 h. However, the protein levels of phospho-IκBα were not obviously increased in these two groups (Fig. 3A–D). Nevertheless, the protein levels of phospho-IκBα were the highest in poly (I: C)-imiquimod-inactivated PRRSV antigen group at 4 h, 8 h and 12 h compared with other groups, respectively (Fig. 3A–D). In the nucleus, the protein levels of NF-κB P65 were gradually increased from 1 h to 12 h in poly (I: C)-imiquimod-inactivated PRRSV antigen group, but not obviously changed in the other groups (Fig. 3A–D). We predicted that TRIF/MyD88-NF-κB signaling pathway was activated in MoDCs treated with poly (I: C), imiquimod along with inactivated PRRSV antigen.

### NF-κB mediated Th1- and Th2-type cytokine secretions in MoDCs treated with TLR3 and 7 ligands along with inactivated PRRSV antigen

**Figure 4:**
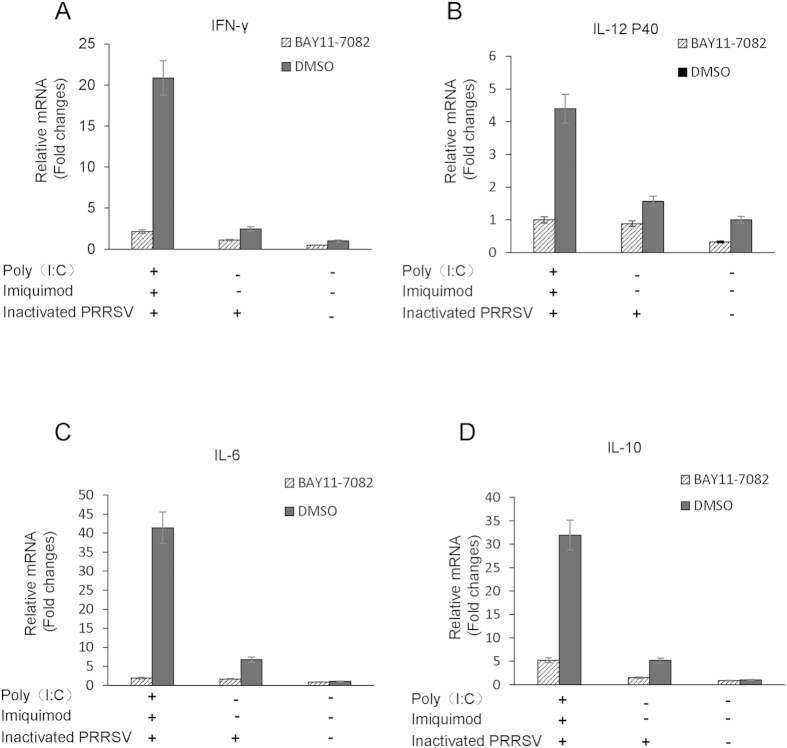
The mRNA levels of cytokines in MoDCs pretreated with NF-κB inhibitor BAY11-7082 or DMSO and then stimulated with poly (I: C) and imiquimod along with inactivated PRRSV antigen. MoDCs were pretreated with BAY11-7082 (5 μM) or DMSO for 2 h, then were incubated with poly (I: C) and imiquimod along with inactivated PRRSV antigen for 12 h, the inactivated PRRSV antigen and RPMI-1640 were used as control. The transcript levels of IFN-γ, IL-12 P40, IL-6 and IL-10 in MoDCs were analyzed by real-time RT-PCR. Relative transcript levels are shown as fold changes relative to the level for the control cells of the RPMI-1640 group. The data represent the means of three independent experiments, with each experiment performed in triplicate. Data represent the means ± standard deviations (error bars) of three independent experiments.

To further investigate the role of NF-κB for regulation of Th1- and Th2-type cytokine secretions in MoDCs treated with TLR3 and 7 ligands along with inactivated PRRSV antigen, MoDCs were pretreated with a specific NF-κB inhibitor BAY11-7082 (5 μM) or DMSO as control for 2 h, cells were then treated with poly (I: C) and imiquimod along with inactivated PRRSV antigen for 12 h. Cells were harvested and the mRNA levels of cytokines were analyzed by real time RT-PCR. As shown in Fig. 4, the mRNA levels of Th1-type cytokines IFN-γ and IL-12 P40, Th2-type cytokines IL-6 and IL-10 were all significantly decreased in MoDCs pretreated with BAY11-7082 in poly (I: C)-imiquimod-inactivated PRRSV antigen group.

### Enhancement of phagocytosis of MoDCs treated with TLR3 and 7 ligands along with inactivated PRRSV antigen

**Figure 5:**
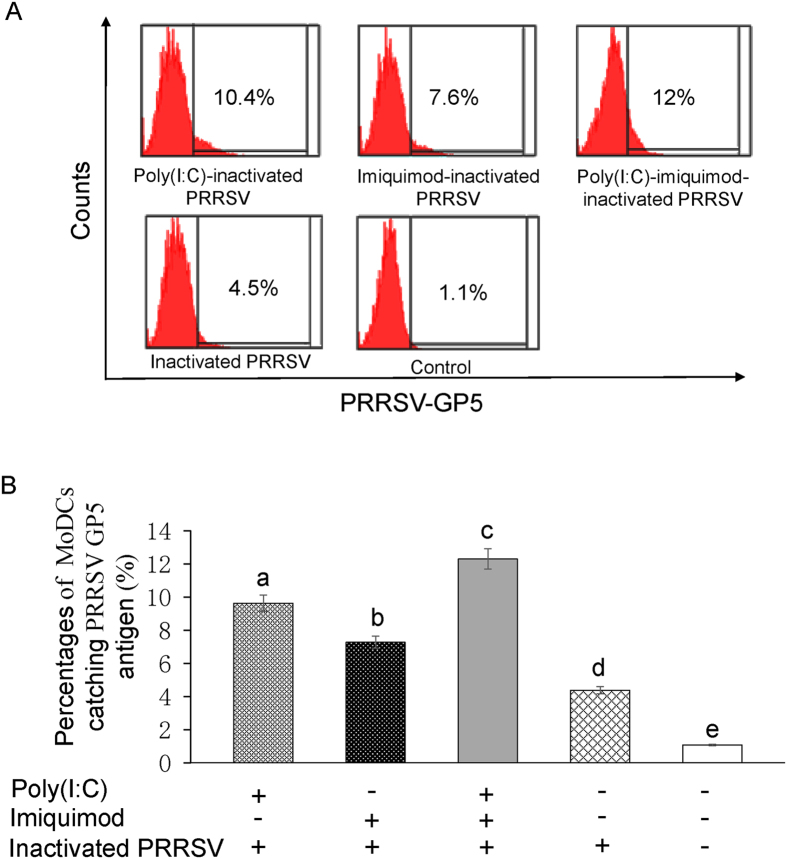
Phagocytosis of MoDCs treated with TLR3 and 7 ligands along with inactivated PRRSV antigen. MoDCs were incubated with poly (I: C) and/or imiquimod along with inactivated PRRSV antigen for 12 h. MoDCs were fixed and permeabilized and then stained with monoclonal antibody against PRRSV GP5. After washing with PBS, cells were incubated with Alexa Fluor 488-conjugated goat anti-mouse IgG(H + L) for flow cytometry. (**A**) Representative flow cytometry profile of the percentages of MoDCs catching PRRSV GP5 antigen. The data presented here are results from one experiment of three flow cytometry experiments. (**B**) The statistical graph of the percentages of MoDCs catching PRRSV GP5 antigen. The data were analyzed using Flowjo7.6 software. Data represent the means ± standard deviations (error bars) of three independent experiments. Different letters (a–e) mean significant difference (*P* < 0.05).

Flow cytometry was conducted to evaluate the capability of MoDCs to phagocytose PRRSV antigen. The positive rates of MoDCs catching PRRSV GP5 antigen in poly (I: C)-inactivated PRRSV antigen group, imiquimod-inactivated PRRSV antigen group, poly (I: C)-imiquimod-inactivated PRRSV antigen group, inactivated PRRSV antigen and RPMI-1640 mock control group were 10.4%, 7.6%, 12.0%, 4.5% and 1.1%, respectively (Fig. 5A). The experiment was repeated 3 times and the data were presented in Fig. 5B. The results suggested that poly (I: C) or imiquimod could enhance the phagocytosis of MoDCs to catch PRRSV antigen, and the combination of poly (I: C) and imiquimod showed the best effect (*P* < 0.05).

### PRRSV-specific T lymphocyte proliferation and percentages of CD4^+^, CD8^+^ T lymphocytes are increased in mice immunized with TLR3 and 7 ligands along with inactivated PRRSV antigen

**Figure 6:**
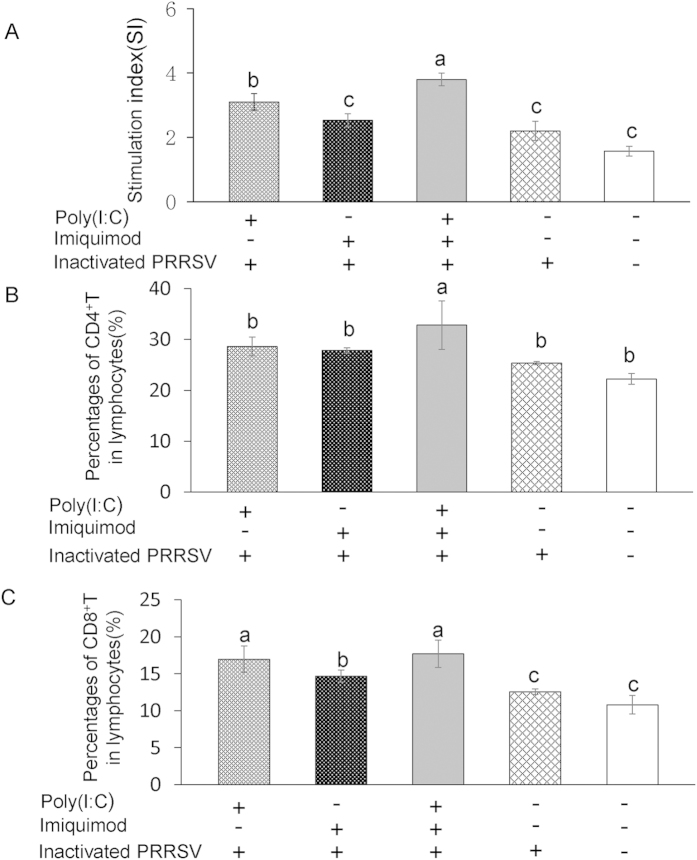
Proliferation of PRRSV-specific T lymphocytes and percentages of CD4^+^, CD8^+^ T lymphocytes in immunized mice. Splenic lymphocytes were isolated from the spleen of vaccinated mice at 49 dpi. Part of splenic lymphocytes was stimulated with purified inactivated PRRSV antigen. Following 72 h incubation, the proliferation response was detected by a standard MTT assay. The PHA control sample showed a stimulation index of 5–7 (**A**). Another part of splenic lymphocytes was subjected to flow cytometry to assess the percentages of CD4^+^ T lymphocytes (**B**) and CD8^+^ T lymphocytes (**C**). Data represent the means ± standard deviations (error bars) of three independent experiments. Different letters (a–c) mean significant difference (*P* < 0.05).

PRRSV-specific T lymphocyte proliferation from splenocytes collected from immunized mice was first detected by MTT assays. As shown in Fig. 6A, the average SI in poly (I: C)-inactivated PRRSV antigen group, imiquimod-inactivated PRRSV antigen group, poly (I: C)-imiquimod-inactivated PRRSV antigen group, inactivated PRRSV antigen and PBS mock control group were 3.1, 2.53, 3.8, 2.2 and 1.57, respectively. The SI was significantly increased in poly (I: C)-inactivated PRRSV antigen group and poly (I: C)-imiquimod-inactivated PRRSV antigen group, and the differences were statistically significant between them (*P* < 0.05). The percentages of CD4^+^, CD8^+^ T lymphocytes in splenocytes were also analyzed by flow cytometry. As shown in Fig. 6B,C, except for CD8^+^ T lymphocytes in poly (I: C)-inactivated PRRSV antigen and poly (I: C)-imiquimod-inactivated PRRSV antigen groups, percentages of both CD4^+^ and CD8^+^ T lymphocytes in splenocytes of mice immunized with poly (I: C)-imiquimod-inactivated PRRSV antigen were significantly increased when compared with the other groups (*P* < 0.05).

### Percentages of PRRSV-specific CD3^+^ T cells producing IFN-γ and IL-4 are augmented in mice immunized with TLR3 and 7 ligands along with inactivated PRRSV antigen

**Figure 7:**
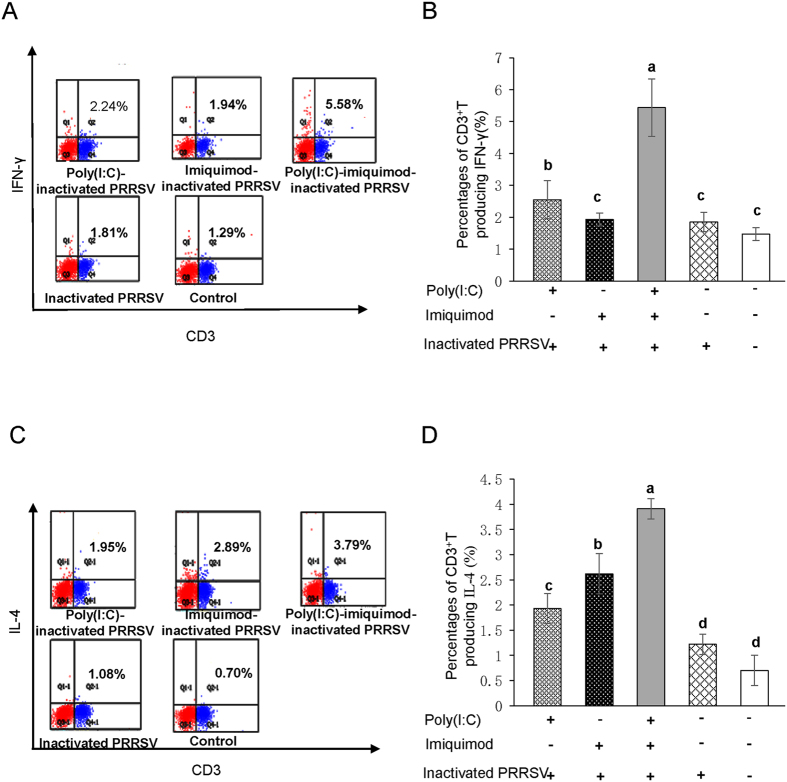
Percentages of PRRSV-specific CD3^+^ T cells producing IFN-γ and IL-4 in immunized mice. DCs were isolated from spleen of mice and stimulated with inactivated PRRSV antigen for 12 h, then DCs were co-cultured with sorted autologous CD3^+^ T cells. Percentages of CD3^+^T cells producing IFN-γ and IL-4 were detected by flow cytometry. (**A**) Representative flow cytometry profile of the percentages of CD3^+^ T cells producing IFN-γ. The data presented here are results from one experiment of three flow cytometry experiments. (**B**) The statistical graph of the percentages of CD3^+^ T cells producing IFN-γ. (**C**) Representative flow cytometry profile of the percentages of CD3^+^ T cells producing IL-4. The data presented here are results from one experiment of three flow cytometry experiments. (**D**) The statistical graph of the percentages of CD3^+^ T cells producing IL-4. The data were analyzed using Flowjo7.6 software. Data represent the means ± standard deviations (error bars) of three independent experiments. Different letters (a–d) mean significant difference (*P* < 0.05).

To assess the effect of DCs from immunized mice on the magnitude of PRRSV-specific T cell responses, the intracellular cytokine production of CD3^+^ T cells was determined by flow cytometry. The percentages of CD3^+^ T cells producing IFN-γ in poly (I: C)-inactivated PRRSV antigen group, imiquimod-inactivated PRRSV antigen group, poly (I: C)-imiquimod-inactivated PRRSV antigen group, inactivated PRRSV antigen and PBS mock control group were 2.24%, 1.94%, 5.58%, 1.81% and 1.29%, respectively. Meanwhile, the percentages of CD3^+^ T cells producing IL-4 were 1.95%, 2.89%, 3.79%, 1.08% and 0.70%, respectively (Fig. 7A,C). The experiment was repeated 3 times and the data were shown in Fig. 7B,D. DCs from mice immunized with poly (I: C)-inactivated PRRSV tended to induce CD3^+^ T lymphocytes producing IFN-γ (*P* < 0.05, Fig. 7A,B). DCs from mice immunized with imiquimod-inactivated PRRSV tended to induce CD3^+^ T lymphocytes producing IL-4 (*P* < 0.05, Fig. 7C,D). Importantly, the highest percentages of CD3^+^ T cells producing IFN-γ and IL-4 were detected in poly (I: C)-imiquimod-inactivated PRRSV antigen group (*P* < 0.05, Fig. 7A–D).

### Levels of Th1- and Th2-type cytokines and neutralization antibody titers are increased in mice immunized with TLR3 and 7 ligands along with inactivated PRRSV antigen

**Figure 8:**
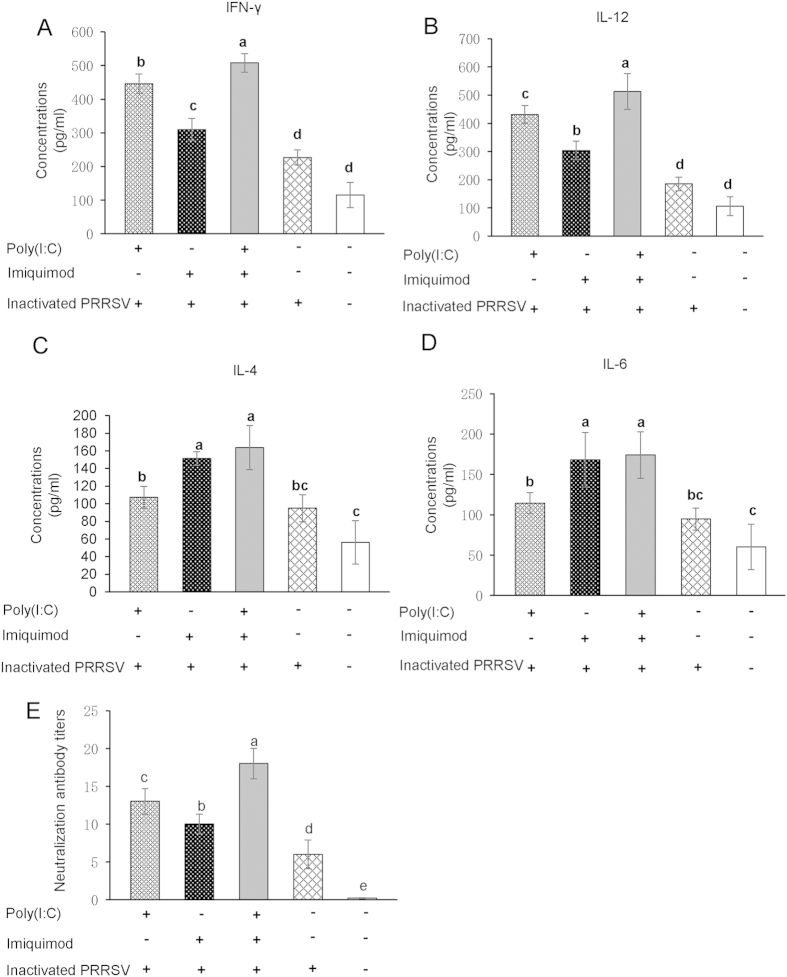
The concentrations of Th1- and Th2-type cytokines and neutralization antibody titers in the sera of immunized mice. Serum samples were collected from vaccinated mice at 49 dpi. The concentrations of Th1-type cytokines IFN-γ and IL-12, Th2-type cytokines IL-4 and IL-6 in the sera were detected by ELISA (**A–D**). Neutralization antibody titers were expressed as the reciprocal of the last serum dilution to neutralize 100 TCID_50_ of PRRSV in 50% of the wells (**E**). Data were shown as means ± standard deviations (error bars). Different letters (a–e) mean significant difference (*P* < 0.05). If there is one same letter, for example, bc and c, it means no significant difference (*P* > 0.05).

The levels of Th1-type cytokines IFN-γ and IL-12, and Th2-type cytokines IL-4 and IL-6 in the sera of immunized mice were measured by ELISA. As shown in Fig. 8A–D, except for the poly (I: C)-imiquimod-inactivated PRRSV antigen group, the higher concentrations of Th1-type cytokines were detected in poly (I: C)-inactivated PRRSV group and Th2-type cytokines were detected in imiquimod-inactivated PRRSV group (*P* < 0.05). The sera were also used to detect the PRRSV-specific neutralization antibody. There was a gradual increase of neutralization antibody titers from inactivated PRRSV antigen group, imiquimod-inactivated PRRSV antigen group, poly (I: C)-inactivated PRRSV antigen group to poly (I: C)-imiquimod-inactivated PRRSV antigen group. The differences among these groups were statistically significant (*P* < 0.05, Fig. 8E).

## Discussion

TLRs express in APCs and other immune cells, and play a central role in controlling innate and adaptive immune responses after exposure to infectious pathogens. Previous studies showed that PRRSV infection could result in a differential expression of TLRs^24^,^25^ and mRNA levels of TLR3, TLR4 and TLR7 were increased in the tracheobronchial lymph nodes or PAMs in PRRSV-infected pigs^25^. Moreover, activating the TLR3 signaling pathway using poly (I: C), the viral load of PRRSV underwent a significant reduction. While the TLR3 expression was suppressed, the PRRSV infectivity was increased^24^,^31^. It has been reported that TLR7 ligand CL097 enhanced the protective effects of vaccination against PRRSV in swine^7^. DCs are potent APCs playing a key role in the induction and regulation of immune response^5^,^23^. To investigate the immunoregulatory effect of TLR ligands on inactivated PRRSV antigen, MoDCs were stimulated with poly (I: C) and/or imiquimod along with inactivated PRRSV antigen *in vitro*. Mice were further used to confirm the immune enhancing effects *in vivo*.

TLR signals could be activated by TLR ligands, which could enhance the maturation of APCs typically accompanied by the increased CD4^+^ and CD8^+^ T cell responses and the robust production of cytokines^32^,^33^,^34^. TLR3 signals could be activated by poly (I: C) followed by subsequent recruitment of TRIF to the receptor, which induces proinflammatory cytokines via TRIF-RIP1/TRAF6-NF-κB pathway. TLR7 signals could be activated by imiquimod or resiquimod and this event leads to engagement of MyD88 to its receptor, which induces proinflammatory cytokines via MyD88-IRAK-4/TRAF6-NF-κB pathway^8^. We showed that the mRNA and the protein levels of Th1-type cytokines IFN-γ and IL-12 P40 (or IL-12) were increased in MoDCs when stimulated with poly (I: C)-inactivated PRRSV antigen, and Th2-type cytokines IL-6 and IL-10 were increased when treated with imiquimod-inactivated PRRSV antigen. Notably, we found that the mRNA and protein levels of Th1- and Th2-type cytokines were the highest in MoDCs when stimulated with poly (I: C)-imiquimod-inactivated PRRSV antigen (Fig. 1), suggesting the synergistic effects of poly (I: C) and imiquimod on MoDCs presenting PRRSV antigen. By the way, Th2-type cytokine IL-4 expressed in MoDCs was very low and hard to be detected, so IL-6 and IL-10 were chosen to detect in our study as reported^35^,^36^.

NF-κB is a transcription factor that regulates the expression of a large number of genes including those involved in inflammation. NF-κB also plays an essential role in innate immune response and many signaling pathways are associated with NF-κB^33^,^36^,^37^,^38^. In previous studies, TRIF mediated innate immune responses in peritoneal mesothelial cells through TLR3 and TLR4 stimulation via activation of NF-κB or MAPKs^39^. Hepatitis C virus NS3 mediated microglial inflammation via TLR2/TLR6-MyD88/NF-κB pathway^40^. Activation of NF-κB via endosomal TLR7 or TLR9 suppressed the reactivation of herpesvirus^17^. It has been demonstrated that TLR2, TLR6, TLR7 and TLR9 contributed to the NF-κB-dependent secretion of TNF in response to Myxoma virus infection^41^. To further clarify the mechanism of synergistic effect of poly (I: C) and imiquimod on MoDCs, TRIF/MyD88-NF-κB signaling pathway was investigated. The results indicated that the mRNA levels of TRIF, MyD88 and NF-κB P65, the cytoplasmic protein levels of TRIF, MyD88, phospho-IκBα and the nuclear protein level of NF-κB P65 in MoDCs were all increased in poly (I: C)-imiquimod-inactivated PRRSV antigen group as early as 8 h after treatment (Figs 2 and 3). However, only TRIF in poly (I: C)-inactivated PRRSV antigen group or MyD88 in imiquimod-inactivated PRRSV antigen group was stimulated. NF-κB P65 was not stimulated in these two groups (Figs 2 and 3).

NF-κB signaling pathway can be inhibited by BAY11-7082 through suppressing IκBα phosphorylation. After inhibiting the activity of NF-κB, the mRNA levels of IFN-γ, IL-12, IL-6 and IL-10 were decreased significantly in poly (I: C)-imiquimod-inactivated PRRSV antigen group, indicating that the production of Th1- and Th2-type cytokines induced by TLR3 and 7 ligands along with inactivated PRRSV antigen was through NF-κB signaling pathway in MoDCs (Fig. 4).

It is reported that activation of TLRs could trigger faster immune responses and the ability of antigen capture is enhanced via TLR-induced actin remodeling^42^. Our data showed that after treatment with poly (I: C) or imiquimod, the ability of capturing inactivated PRRSV antigen of MoDCs was enhanced. The combination of poly (I: C) and imiquimod showed the best enhancing effect (Fig. 5). Such a combination is likely to help MoDCs to trigger faster and stronger immune responses. From the results of MoDCs, we speculate that the synergistic effect of poly (I: C) and imiquimod on presenting inactivated PRRSV antigen is through TLR/MyD88-NF-κB signaling pathway.

Activated CD4^+^ T cells can be classified into at least two subgroups, Th1 and Th2. The Th1-like phenotype, predominantly associated with IL-2, IL-12 and IFN-γ, is a hallmark of the cellular immune response. The Th2-like phenotype such as IL-4, IL-5, IL-6, IL-8 and IL-10 is indicative of the humoral and mucosal immune response^43^,^44^,^45^. In our experiment, the PRRSV-specific T lymphocyte proliferation and the percentages of CD4^+^ and CD8^+^ T lymphocytes in splenocyte were increased in mice immunized with poly(I: C)-imiquimod-inactivated PRRSV antigen (Fig. 6). Moreover, the percentages of PRRSV-specific CD3^+^ T cells producing IFN-γ and IL-4, the levels of Th1- and Th2-type cytokines were also augmented (Figs 7 and 8A–D), suggesting the cellular and humoral immune responses are significantly enhanced by co-stimulation of poly (I: C) and imiquimod. The results of neutralization antibody of sera from mice immunized with poly (I: C)-imiquimod-inactivated PRRSV antigen further confirmed that humoral immune response was enhanced (Fig. 8E).

Recently, it was reported that TLR ligands and the combination of TLR ligands could modulate DCs to augment antigen-specific T cells response against some viral diseases and tumors in humans^34^,^45^,^46^,^47^,^48^,^49^,^50^,^51^,^52^,^53^. The combination of TLR ligands were also used as vaccine adjuvant for their stronger adjuvanticity and immunity^54^. However, the effect of the combination of TLR3 and 7 ligands on inactivated PRRSV antigen has not been explored. In this study, we first elucidated the immune enhancing effect and the mechanism of the combination of TLR3 and 7 ligands on inactivated PRRSV antigen in MoDCs and mice. *In vitro*, the combination of TLR3 and 7 ligands can significantly enhance the mRNA and protein levels of Th1- and Th2-type cytokines, and the ability of presenting inactivated PRRSV antigen in MoDCs through TRIF/MyD88-NF-κB signaling pathway. *In vivo*, the combination of TLR3 and 7 ligands significantly enhances cell-mediated and humoral immune responses of inactivated PRRSV antigen in mice. Our results provide novel insights to the potency of the combination of TLR3 and 7 ligands in effective stimulation of inactivated PRRSV antigen presentation, which can be further explored in PRRS inactivated vaccine development.

## Methods

### Cells and viruses

MARC-145 cells were grown in Dulbecco’s modified Eagle’s medium (DMEM) supplemented with 10% fetal bovine serum (FBS; HyClone, Logan, UT, USA), 2 mM L-glutamine, 100 U penicillin/ml and 100 μg streptomycin/ml in a humidified incubator with 5% CO_2_ at 37 °C. The 10^th^ passage of HP-PRRSV SD-JN strain was propagated and titrated in MARC-145 cells with 1 × 10^7.0^ TCID_50_/ml. The virus was then inactivated by incubating with 0.05% β-propiolactone (Ferak Berlin Gmbh, Berlin, Germany) at 4 °C for 12 h followed by additional 2 h at 37 °C. The inactivated virus was tested for the residual infectivity by blindly passaging three times in MARC-145 cells. Neither CPEs nor viral genome was detected, indicating the complete inactivation by the chemical agent and procedure above. The SD-JN HP-PRRSV whole virion antigen was purified and quantitated by optical density (OD) measurement as described previously^55^.

### Preparation and stimulation of MoDCs

Blood samples were collected from 6-week-old healthy crossbred pigs, which were obtained from a local farm without PRRSV, porcine circovirus 2 (PCV-2), porcine parvovirus (PPV), pseudorabies virus (PRV) and Actinobacillus pleuropneumoniae (APP) history. All pigs were tested and proven to be seronegative for PRRS by indirect enzyme-linked immunosorbent assay (iELISA) and PRRSV negative by RT-PCR. Peripheral blood mononuclear cells (PBMCs) were isolated following centrifugation (1000 × *g* for 30 min) over Ficoll-Paque PLUS (d = 1.007, GE Healthcare, Uppsala, Sweden). Cells were then washed three times with PBS to remove platelets and cell debris. Subsequently, PBMCs were resuspended in RPMI-1640 medium and then plated in six-well plates at a density of 1 × 10^7^/ml and incubated for 2 h at 37 °C with 5% CO_2_. After washing twice gently to remove non-adherent cells, adherent cells were cultured in RPMI-1640 medium containing 10% FBS and stimulated with 10 ng/ml rpIL-4 (R&D systems, Inc., Minneapolis, USA), 20 ng/ml rpGM-CSF (R&D systems) at 37 °C with 5% CO_2_ for 6 days to make cells differentiate into MoDCs. Half of culture medium was removed with the replacement by equal volume of fresh medium every two days. 6 days later, single or clustered non-adherent, veiled-shaped cells were observed. Percentages of cells expressing MHC II and B7 were detected by flow cytometry and used for evaluating the differentiation^56^, which were gradually increased with the time and reached 61.5% MHC II and 59% B7 at 6 days.

MoDCs were cultured in six-well plates and divided into five groups: poly (I: C)-inactivated PRRSV antigen group, imiquimod-inactivated PRRSV antigen group, poly (I: C)-imiquimod-inactivated PRRSV antigen group, inactivated PRRSV antigen group and RPMI-1640 group as mock control. For groups 1–3, MoDCs were treated with poly (I: C) (20 μg/ml) (Invivogen, San Diego, CA) and/or imiquimod (5 μg/ml) (Invivogen) along with 1 × 10^6.0^ TCID_50_ inactivated PRRSV antigen per well. For group 4, MoDCs were treated with 1 × 10^6.0^ TCID_50_ inactivated PRRSV antigen per well. MoDCs in group 5 were cultured with RPMI-1640 and used as mock control.

### Immunization of BALB/c mice

The animal experiments were approved by Shandong Provincial Science and Technology department in China and conducted accordingly. Experiments conformed to the local (Regulations for the administration of affairs concerning experimental animals) and international (Dolan K. 2007 Second Edition of Laboratory Animal Law. Blackwell, UK) guidelines on the ethical use of animals. Fifty 6-week-old female BALB/c mice (provided by animal experiment center of Shandong University, Jinan, China) were randomly divided into five groups: poly (I: C)-inactivated PRRSV antigen group, imiquimod-inactivated PRRSV antigen group, poly (I: C)-imiquimod-inactivated PRRSV antigen group, inactivated PRRSV antigen group and PBS group as mock control. For groups 1–3, mice were vaccinated with 20 μg of poly (I: C) and/or 5 μg of imiquimod along with 1 × 10^6.0^ TCID_50_ inactivated PRRSV antigen. For group 4, mice were vaccinated with 1 × 10^6.0^ TCID_50_ inactivated PRRSV antigen. Mice in group 5 were injected with PBS and used as mock control. All groups of mice were injected subcutaneously twice at a 3 week interval. At 49 days post primary immunization (dpi), all mice were euthanized and the sera were harvested for the detection of antibodies against PRRSV using serum neutralization (SN) assay. These samples were also assayed for Th1- and Th2-type cytokines by ELISA. Meanwhile, the lymphocytes were separated from the spleen of each mouse. One part of splenic lymphocytes was used for detection of PRRSV-specific T lymphocyte proliferation and the percentages of CD4^+^, CD8^+^ T lymphocytes, while the other part of splenic lymphocytes was used for further evaluation of the percentages of PRRSV-specific CD3^+^ T cells producing IFN-γ and IL-4.

### Real-time PCR

To determine the mRNA levels of cytokines in MoDCs, the total RNA of MoDCs was extracted using TRIzol reagent (Invitrogen, Carlsbad, CA, USA) according to the manufacturer’s protocol. 1 μg RNA was reversed transcribed using Primer Script^TM^ RT Reagent Kit with gDNA Eraser (Perfect Real Time) (Takara Co., Ltd., Japan). 2 μl of 20 μl cDNA was subjected to SYBR green PCR using the Roche LightCycle^®^ 480 II sequence detection system and analyzed with the Roche LightCycle^®^ 480 II software. Real-time PCR primers used in this study were listed in Table 1. The abundance of individual mRNA transcript in each sample was assayed three times and normalized to that of β-actin mRNA (as an internal control). Relative transcript levels were quantified by the 2^−ΔΔCt^ (where Ct is threshold cycle) method and shown as fold changes relative to the level for the control cells of the RPMI-1640 group.

### Western blotting assay

The nuclear fraction was extracted from MoDCs using a nuclear/cytosol fractionation kit according to the manufacturer’s instructions (BioVision, Mountain View, CA, USA). The samples were analyzed by sodium dodecyl sulfate-polyacrylamide gel electrophoresis (SDS-PAGE) and western blotting analysis. Briefly, the cytoplasmic or nuclear fractions were resolved in a 10% polyacrylamide gel. Separated proteins were then transferred onto a PVDF transfer membrane and probed with antibody against TRIF (Novus, Littleton, USA), MyD88 (Abcam, Cambridge, UK), phospho-IκBα (Cell Signaling, Danvers, MA, USA), NF-κB P65 (Cell Signaling), β-actin (Santa Cruz Biotechnology, Santa Cruz, CA, USA) or PCNA (Novus), respectively. Specific reaction products were detected with horseradish peroxidase (HRP)-conjugated goat anti-mouse IgG (Boster, Wuhan, China). Signals were captured and measured using the Molecular Imager^®^ ChemiDoc^TM^ XRS^+^ systems with Image Lab^TM^ software (Bio-Rad).

### ELISA

Concentrations of Th1- and Th2-type cytokines in the supernatants of MoDCs or in the sera of mice were measured using commercially available ELISA kits following the manufacturer’s instructions (R&D systems). Each sample was analyzed three times independently.

### Isolation of lymphocytes, DCs and CD3^+^ T cells from spleens of mice

Splenic lymphocytes were collected and cultured as described previously^55^. Part of splenic lymphocytes was incubated with anti-CD11c microbeads (Miltenyi Biotec, Bergisch Gladbach, Germany) and separated using AutoMacs (Miltenyi Biotec). Selected cells were considered CD11c^+^ DCs. Negatively selected cells were stained with anti-CD3 FITC (Miltenyi Biotec), and subsequently incubated with anti-FITC microbeads (Miltenyi Biotec) to obtain CD3^+^ T cells. DCs and CD3^+^ T cells were cultured in RPMI-1640 medium supplemented with 10% FBS for further detection of the percentages of PRRSV-specific CD3^+^ T cells producing IFN-γ and IL-4.

### Flow cytometry analysis

To determine the function of MoDCs to phagocytose inactivated PRRSV antigen, MoDCs were fixed and permeabilized using Cytofix/Cytoperm according to the manufacturer’s instructions (BD Biosciences, San Jose, USA). MoDCs were then stained with monoclonal antibody against PRRSV GP5 (kindly provided by Dr. Guangzhi Tong, Shanghai Veterinary Research Institute, Chinese Academy of Agricultural Sciences, Shanghai, China) at 4 °C for 1 h. After washing with PBS, cells were incubated with Alexa Fluor 488-conjugated goat anti-mouse IgG(H + L) (Invitrogen) in the dark for 1 h, washed twice with PBS, and resuspended in PBS for flow cytometry (FACSAria III, BD Biosciences). The data were analyzed using the Flowjo7.6 software.

In order to evaluate the percentages of CD4^+^ T and CD8^+^ T cells in splenic lymphocytes of mice, the isolated splenic lymphocytes were incubated at 4 °C with anti-CD4 PE (Miltenyi Biotec) and anti-CD8 APC (Miltenyi Biotec) in the dark for 30 min. The expression of the different cell surface markers was analyzed by flow cytometry as described above.

For detection of the percentages of PRRSV-specific CD3^+^ T cells producing IFN-γ and IL-4, the isolated DCs from spleen of mice were stimulated with 1 × 10^6.0^ TCID_50_ inactivated PRRSV antigen for 12 h. Purified autologous CD3^+^ T cells were added to the DC cultures at a ratio of 1 DC per 10 CD3^+^ T cells, and incubated for 4 h at 37 °C with 5% CO_2_, followed by an additional 4 h in the presence of the secretion inhibitor leukocyte activation cocktail with BD GolgiPlug^TM^ (10 μg/ml; BD Pharmingen^TM^, USA). The co-cultured DC-T cells were washed, stained with anti-CD3 FITC, fixed and permeabilized using Cytofix/Cytoperm, and then stained with anti-IFN-γ PE (Miltenyi Biotec) and anti-IL-4 APC (Miltenyi Biotec) in the dark for 30 min. The cells were washed twice and analyzed by flow cytometry as described above.

### T lymphocyte proliferation assay

Splenic lymphocytes obtained as above were resuspended to 5 × 10^6^/ml in RPMI-1640 medium supplemented with 10% FBS and seeded in 96-well flat-bottom plates at 100 μl per well. Each cell sample was plated in triplicate. The cells were stimulated with purified SD-JN HP-PRRSV antigen at a final concentration of 10 μg/ml or unstimulated, respectively. Meanwhile, phytohemagglutinin (PHA) was used as a positive control (10 μg/ml; Sigma-Aldrich, St. Louis, MO, USA). After incubation for 72 h at 37 °C with 5% CO_2_, the proliferation responses were detected by a standard MTT method. T lymphocyte proliferation was expressed as stimulation index (SI), which is the ratio of OD_570_ _nm_ of stimulated well to that of unstimulated one^26^,^55^.

### SN assays

SN assays were performed as previously described^55^. All serum samples from mice were heat inactivated (56 °C, 30 min) and 1:2 serially diluted. Then, the serial dilutions of serum were mixed with equal volumes of 200 TCID_50_ HP-PRRSV SD-JN strain. After incubation at 37 °C for 1 h, the mixtures were transferred to MARC-145 monolayers in 96-well tissue culture plates. The plates were incubated and observed daily for up to 5 days for the appearance of CPE. Meanwhile, PRRSV positive and negative sera of mice (kept in our lab) were used as positive and negative controls, respectively. CPE was used to determine the end-point titers that were calculated as the reciprocal of the last serum dilution to neutralize 100 TCID_50_ of PRRSV in 50% of the wells.

### Statistical analysis

Data were compared and the differences were determined by One-way repeated measurement ANOVA and Least significance difference (LSD). A *P*-value < 0.05 was considered statistically significant^55^.

## Additional Information

**How to cite this article**: Hu, Y. *et al.* Synergy of TLR3 and 7 ligands significantly enhances function of DCs to present inactivated PRRSV antigen through TRIF/MyD88-NF-κB signaling pathway. *Sci. Rep.* **6**, 23977; doi: 10.1038/srep23977 (2016).

## Figures and Tables

**Table 1:** Primers used in the study.

Primer name	Sequence (5′–3′)	Purpose
IFN-γ-Fwd	GGAGCATGGATGTGATCAAG	IFN-γ amplification
IFN-γ-Rev	GAGTTCACTGATGGCTTTGC	
IL-12 P40-Fwd	GGGTGGGAACACAAGAGAT	IL-12 P40 amplification
IL-12 P40-Rev	GGCTAAACTTGCCTAGAGGT	
IL-6-Fwd	TGGGTTCAATCAGGAGACCT	IL-6 amplification
IL-6-Rev	CAGCCTCGACATTTCCCTTA	
IL-10-Fwd	TGCTCTATTGCCTGATCTTCCTG	IL-10 amplification
IL-10-Rev	AGTCGCCCATCTGGTCCTTC	
TRIF-Fwd	CACCTTCTGCGAGGATTTC	TRIF amplification
TRIF-Rev	GCTGCTCATCAGAGACTGGT	
MyD88-Fwd	GGAACAGACCAACTATCGGC	MyD88 amplification
MyD88-Rev	GAGACAACCACTACCATCCG	
NF-κB P65-Fwd	GGGACTACGACCTGAATGCT	NF-κB P65 amplification
NF-κB P65-Rev	GGGCACGGTTGTCAAAGAT	
β-actin-Fwd	TCTGGCACCACACCTTCT	β-actin amplification
β-actin -Rev	GATCTGGGTCATCTTCTCAC	
